# Risk Assessment of Infectious Endogenous Banana Streak Viruses in Guadeloupe

**DOI:** 10.3389/fpls.2022.951285

**Published:** 2022-07-11

**Authors:** Marie Umber, Gersende Pressat, Guillaume Fort, Kaïssa Plaisir Pineau, Chantal Guiougiou, Frédéric Lambert, Benoît Farinas, Jean-Philippe Pichaut, Bérenger Janzac, Jean-Marie Delos, Frédéric Salmon, Cécile Dubois, Pierre-Yves Teycheney

**Affiliations:** ^1^CIRAD, UMR AGAP Institute, Guadeloupe, France; ^2^UMR AGAP Institute, University of Montpellier, CIRAD, INRAE, Institute Agro, Guadeloupe, France; ^3^CIRAD, UMR AGAP Institute, Montpellier, France; ^4^UMR AGAP Institute, University of Montpellier, CIRAD, INRAE, Institute Agro, Montpellier, France

**Keywords:** endogenous banana streak viruses, infectious alleles, activation, *Musa*, risk assessment

## Abstract

Infectious alleles of endogenous banana streak viruses (eBSVs) are present in the genome of all banana interspecific cultivars, including plantains and cooking types. Activation of these infectious eBSV alleles by biotic and abiotic stresses leads to spontaneous infections by cognate viruses and raises concerns about their ability to promote outbreaks of banana streak viruses under field cultivation conditions. We undertook a comprehensive risk assessment study of infectious eBSV alleles of species BSOLV, BSGFV and BSIMV in banana interspecific cultivars in Guadeloupe, a tropical island of the Caribbean where bananas are grown for export and local markets. We carried out a prevalence survey of BSOLV, BSGFV and BSIMV species in a range of cultivars grown in Guadeloupe. Our results suggest that BSOLV and BSGFV infections arise from the activation of infectious eBSVs rather than vector-borne transmission and point to a correlation between altitude and infection rates in interspecific hybrids with AAB genotypes. We studied the dynamics of activation of infectious eBSOLV and eBSGFV alleles by tissue culture and field cultivation in a range of cultivars. We showed that tissue culture and field cultivation trigger distinct activation pathways, resulting in distinct activation patterns. We also showed that activation decreased over time during cell culture and field cultivation and that BSV infections arising from the activation of infectious eBSV alleles cause symptomless infections in the most cultivated plantain in Guadeloupe, French Clair. Overall, our study shows that the risk of BSV outbreaks resulting from the activation of infectious eBSVs in plantain originating from vegetative multiplication is negligible in Guadeloupe.

## Introduction

Banana is one of the major food commodities after rice, wheat and maize. Dessert banana is the most exported fresh fruit worldwide both in volume and value, representing an essential source of income and employment for hundreds of thousands of rural households in developing countries (Scott, 2021). Cooking types and plantains are essentially staple food grown for local consumption and their role in local economies is vital to millions of small-scale farmers. Banana belongs to the genus *Musa* in the family *Musaceae*. Edible bananas, including plantains and other cooking types, arose from combinations of species *Musa acuminata*, whose genome is denoted A, and *M. balbisiana*, whose genome is denoted B (Simmonds, 1962; Perrier et al., 2011). The nuclear genome of all known diploid *Musa balbisiana* genitors harbors infectious endogenous banana streak virus sequences (eBSVs), whose activation by abiotic stresses is associated with spontaneous infections by cognate viruses in diploid AB, triploid AAB and tetraploid AAAB interspecific hybrids (Dallot et al., 2001; Gayral et al., 2008; Côte et al., 2010; Chabannes et al., 2013; Duroy et al., 2016).

Banana streak viruses (BSVs) are mealybug-transmitted members of the genus *Badnavirus* in the family *Caulimoviridae* (Geering, 2021). They infect all types of banana and cause typical symptoms including leaf streaks, pseudostem splitting and cracks in fruit skins (Lockhart and Jones, 2000). Nine banana streak virus species are currently recognized by the International Committee for the Taxonomy of Viruses (ICTV; Teycheney et al., 2020). eBSVs of three of these species, *Banana streak OL virus* (BSOLV), *Banana streak GF virus* (BSGFV) and *Banana streak IM virus* (BSIMV), have been reported in the *M. balbisiana* genome (Chabannes et al., 2013). It was shown that eBSOLV, eBSGFV and eBSIMV are each present at a single locus in the genome of seedy diploid model species Pisang Klutuk Wulung (PKW): eBSOLV and eBSGFV loci each contain two distinct alleles (OL1/OL2 and GF7/GF9, respectively) of which OL1 and GF7 are infectious, whereas the eBSIMV locus has two structurally identical and infectious alleles labeled IM (Chabannes et al., 2013).

Since their discovery in 1999 (Harper et al., 1999; Ndowora et al., 1999), infectious eBSVs have been the major constraint for breeding interspecific hybrids and, to a certain extent, for cultivating such hybrids, due to the risk of BSV outbreaks resulting from mealybug transmission following stress-induced activation (Meyer et al., 2008). Following the characterization of infectious eBSVs in the genome of model species PKW, *M. balbisiana* diploid genitors devoid of infectious eBSV alleles were obtained (Umber et al., 2016), paving the way to the breeding of new banana interspecific hybrid varieties with no risk of activation of infectious eBSVs. However, several widely cultivated banana interspecific cultivars harbor infectious eBSV alleles that can be activated by tissue culture, leading to spontaneous BSV infections (Dallot et al., 2001; Côte et al., 2010).

Banana is one of the major agricultural productions in Guadeloupe. Triploid AAA Cavendish type dessert banana is grown for export, whereas triploid AAB cultivars such as Figue Pomme and plantains of the French group are grown for local markets. All banana types are also grown as subsistence crops in private Creole gardens. The use of vitroplants (VPs) as planting material has been enforced for decades in Guadeloupe for Cavendish-type dessert banana, proving an efficient strategy for the control of pests and pathogens, but not for plantain considering the risk of activation of infectious eBSVs by tissue culture in AAB genotypes (Dallot et al., 2001; Meyer et al., 2008; Côte et al., 2010). However, the risk of BSV outbreaks originating from vegetatively propagated plantain has never been assessed under field conditions, precluding the implementation of proper risk management strategies. Meanwhile, suckers remain the primary source of planting material for plantain types in Guadeloupe, although their non-certified phytosanitary status is problematic because they have the potential to spread pests and bacterial, fungal and viral diseases.

In the present study, we undertook a comprehensive risk assessment study of infectious eBSVs in Guadeloupe spanning a ten-year period. Firstly, the prevalence of viral species BSOLV, BSGFV and BSIMV was assessed by a survey in the main banana types cultivated in Guadeloupe. Our results suggest that BSIMV is not present in Guadeloupe. They show that the prevalence of BSOLV and BSGFV is significantly higher in AAB genotypes than in AAA genotypes, suggesting that infections by these viral species arise from the activation of infectious eBSVs rather than vector-borne transmission. Secondly, the dynamics of activation of infectious eBSVs by tissue culture was assessed in cultivars with AB, AAB, ABB and AABB genotypes over 10 *in vitro* subcultures. Thirdly, activation of infectious eBSVs was monitored under field cultivation conditions over 3 production cycles in two triploid cooking type bananas, plantain French Clair (AAB) and Pelipita (ABB). Our results showed that cell culture and field culture trigger distinct activation pathways of eBSOLV and eBSGFV infectious alleles, resulting in distinct activation patterns but that they do not activate eBSIMV infectious alleles. Our results also showed that activation of eBSOLV and eBSGFV infectious alleles in cultivars French Clair and Pelipita decreases over time during cell culture and under field conditions and that BSV infections cause symptomless infections in these cultivars in Guadeloupe. Overall, our study shows that the risk of BSV outbreaks resulting from the activation of infectious eBSVs in French Clair plants originating from vegetative multiplication is negligible in Guadeloupe.

## Materials and Methods

### Plant Materials

Leaf samples used for the prevalence survey were collected throughout Guadeloupe’s main banana production areas from plots cultivated for commercial production, domestic orchards (Creole gardens) cultivated for private consumption, uncultivated or abandoned plots and roadsides (Supplementary Figure 1 and Supplementary Table 1). The cultivar and genotype of sampled plants, GPS coordinates and altitude of sampling sites, presence of symptoms and mealybugs were recorded for each collected sample (Supplementary Table 1).

Planting material used for the field trial was produced from virus-free plants originating from the Biological Resource Center for Tropical Plants (BRC TP;^1^) by vegetative propagation either by cell culture (vitroplants) or by horticultural multiplication. Vitroplants were produced from virus-free shoots of cultivars French Clair, Pelipita and Flhorban 925 using the *in vitro* budding method described by Côte et al. (1990) and modified as follows. Meristematic buds were placed on 0.5x MS regeneration medium (Dutscher, Bernolsheim, France) supplemented with sucrose (20 g/L), adenine (120 mg/L) and activated charcoal (1 g/L) and placed at 25°C in the dark until buds started to open up. They were then transferred to 0.5x MS proliferation medium (Dutscher, Bernolsheim, France) supplemented with sucrose (20 g/L), adenine (120 mg/L), cysteine (50 mg/L) and 6-benzylaminopurine (BAP; 10 mg/L) and placed at 25°C in the dark until shoots appeared, then under a 16 h of light/8 h of dark photoperiod. Plantlets produced by horticultural multiplication were generated using the stem fragments (PIF) technique developed by Kwa (2003), from virus-free plants of cultivars French Clair, Pelipita and Flhorban 925 originating from the BRC TP. French Clair vitroplants used for the assessment of the activation of French Clair’s infectious alleles OL1 and GF7 under contrasting conditions were produced by Vitropic SA (St Mathieu de Tréviers, France). All produced plantlets were acclimatized in an insect-proof greenhouse until they reached the 3-leaf stage and planted in the field where they were submitted to monthly fertilizer applications (100 g/plant of complete fertilizer, 15-4-30 NPK) and standard cultivation practices including desuckering and bunch care.

### Banana Streak Viruses Indexing and Endogenous Banana Streak Viruses Profiling

Virus indexings were performed according to Le Provost et al. (2006) modified by Umber et al. (2016), using a polyclonal antibody either provided by B.E.L. Lockhart (University of Minnesota, United States) or purchased from Neogen (Ayr, Scotland). eBSV genotyping was performed as described by Umber et al. (2016).

### Kinetics of Activation of Infectious eBSOLV, eBSGFV, and eBSIMV Alleles by Cell Culture

The activation of eBSOLV, eBSGFV and eBSIMV infectious alleles by cell culture was monitored in 12 banana cultivars of various genotypes originating from the germplasm collection of the BRC TP (Supplementary Table 2). For this, vitroplants were produced from virus-free suckers using the *in vitro* budding method of Côte et al. (1990) modified as described above. Three to five meristematic buds were used for each cultivar to initiate the multiplication process, resulting in one to three independent lines per cultivar following the necrosis and death of some meristematic buds at the beginning of the process (Supplementary Table 3). The kinetics of activation of eBSOLV, eBSGFV and eBSIMV infectious alleles was monitored in each line over 10 proliferation stages spanning a 20 months period. For this, up to forty shoots were harvested for each line from proliferation clumps at each subculture step, depending on shoot availability, and splitted in two. One half of each shoot was used for BSV indexing as described above, whereas the other half was placed in vials containing fresh proliferation medium and used to carry on the proliferation process. Half-shoots whose counterparts were indexed positive were discarded from the experiment.

### Kinetics of Activation of Infectious eBSOLV, eBSGFV, and eBSIMV Alleles Under Field Conditions

An experimental trial was established in April 2015 to assess the activation of eBSVOLV, eBSGFV and eBSIMV infectious alleles in varieties French Clair (AAB) and Pelipita (ABB) under field conditions. The plot was located at CIRAD’s Neufchateau outstation in Guadeloupe (altitude 250 m) and comprised a total of 464 plants 2.5 m apart from each other (Supplementary Figure 2), resulting in a density of 1,683 plants per hectare. Five types of planting material were used and each was considered a statistical treatment (T): French Clair vitroplants (FRC*VP; T1), French Clair PIF (FRC*PIF; T2), Pelipita vitroplants (PLP*VP; T3), Pelipita PIF (PLP*PIF; T4); vitroplants of cultivar Flhorban 925 (925*VP; T5), which have AAA genotypes and are devoid of eBSVs, were included in the trial and used as negative controls of activation of infectious eBSV alleles. The experimental plot was designed in 80 randomized complete blocks in which each block contained 5 elementary plots of one plant each. Planting material (T1 to T5) was randomly assigned to each elementary plot within each block (Supplementary Figure 2 and Supplementary Table 4). The plot was surrounded by a border of 64 Flhorban 925 plants originating from vitroplants, which served to monitor possible vector-borne transmission of BSVs from outside the plot. BSV symptoms were monitored and leaf samples were collected from all 464 plants every 3 months during a 24 months period encompassing two to three crop cycles, and used for virus indexing.

An additional set of 9 plots was established in July 2017 in privately owned farms ranging in altitudes between 22 and 275 m (Supplementary Figure 3 and Supplementary Table 5A). Planting material originated from vitroplants of cultivar French Clair produced by Vitropic SA (Saint-Mathieu-de-Tréviers, France). Each plot had a surface ranging from 0.15 ha to 0.22 ha and comprised 277-400 plants at the time of planting, resulting in plant densities comprised between 1767 and 1847 plants per hectare (Supplementary Table 5A). BSV leaf symptoms were monitored every 3 months throughout a 24 months period. Leaf samples were collected from all the plants of the plots at flowering stage at 12 months and 24 months after planting and were used for BSV indexing as described above.

### Statistical Analyses

Contingency tables were computed with the prevalence survey data for cross-referencing the indexing results for each viral species with the most frequent genotypes. Chi-square was calculated on these cross-tables to numerically assess which cells deviate most from the independence hypothesis. When expected numbers were too small for the validity of an asymptotic test, independence was tested by a Fisher’s exact test with simulations of Monte Carlo (10,000 replicates; SAS 9.3 software, proc FREQ).

Logistic regression was implemented to analyze the results of the viral indexings from the randomized block field trial. Since many blocks had zero mean and variance, the block factor was treated as a random factor. Two generalized mixed models were tested in an attempt to circumvent the problems of non-estimability of parameters, due to the large number of null responses (SAS 9.3 software, proc GLIMMIX).

## Results

### Prevalence of BSOLV, BSGFV, and BSIMV in Guadeloupe

A total of 1514 leaf samples were collected throughout Guadeloupe from banana varieties Figue sucrée (AA genotype), Cavendish (AAA genotype), Figue Pomme (AAB genotype), French Clair (AAB genotype), Poteau Géant (ABB genotype), Abacá (TT genotype) and varieties with unknown genotypes (Supplementary Table 1). Considering the low number of collected samples for cultivars with AA (30 samples), TT (3 samples) and unknown (10 samples) genotypes, these 43 samples, which were all non-infected, were not used for statistical analyses, leaving a total of 1471 samples originating from plants with AAA, AAB or ABB genotypes (Table 1) for these analyses. The eBSV patterns of all sampled cultivars were determined (Supplementary Table 2). As expected, cultivars with no B genome were found devoid of all eBSV whereas all other sampled cultivars, which have at least one copy of the B genome, harbored eBSVs. The genome of cultivar French Clair was devoid of eBSIMV (Table 1 and Supplementary Table 2). Some eBSV alleles were considered modified (MOD) when their molecular pattern differed from that of model species Pisang Klutuk Wulung which served to design the molecular markers that were used to characterize eBSV patterns (Chabannes et al., 2013). For example, the genome of cultivar Figue Pomme harbored modified eBSOLV and eBSIMV alleles, and that of cultivar Poteau Géant a modified eBSOLV allele.

**TABLE 1:** Prevalence of BSGFV, BSOLV and BSIMV in the collected samples.

		BSOLV				BSGFV				BSIMV			
Varitiety (genotype)	Plant samples	eBSOLV pattern	Non-infected	Infected	% infected	eBSGFV pattern	Non-infected	Infected	% infected	eBSIMV pattern	Non-infected	Infected	% infected
Cavendish (AAA)	**557**	no eBSOLV	554	**3**	0,5%	no eBSGFV	557	**0**	0,0%	no eBSIMV	557	**0**	0,0%
Figue Pomme (AAB)	**307**	MOD	298	**9**	2,9%	GF9	307	**0**	0,0%	MOD	307	**0**	0,0%
French Clair (AAB)	**446**	OL1	445	**1**	0,2%	GF7	434	**12**	2,7%	no eBSIMV	446	**0**	0,0%
Poteau Géant (ABB)	**161**	MOD	159	**2**	1,2%	GF7 + GF9	160	**1**	0,6%	IM	161	**0**	0,0%
**Total**	**1471**		1456	**15**	1,0%		1458	**13**	0,9%		1471	**0**	0,0%
MC Estimate for the Exact Test^(1)^		0.0027 **				< 0.0001 ***							

(1) P-value for the Fisher’s exact test by Monte Carlo simulations, 10000 replicates. (**, ***: significant at level alpha = 1%, 1‰, respectively. Colors highlight cells with the highest chi-square contributions, for observed counts lower (red) or higher (green) than those expected under the independence hypothesis. GF7, OL1, IM: infectious eBSV alleles. GF9, OL2: non-infectious alleles. MOD: modified allele.

BSIMV could not be detected in any of the analyzed samples, confirming previous findings from a smaller sampling (Péréfarres et al., 2009) and suggesting that this viral species is absent from Guadeloupe. BSIMV is also presumably not present in the nearby Dominican Republic (Martinez et al., 2020) but was reported in Cuba (Javer et al., 2009). Only 28 plants were indexed positive and no mixed infection was reported, resulting in overall prevalence of BSOLV and BSGFV of 1 and 0.9%, respectively. Differences in prevalence were observed between cultivars (Table 1). The low overall prevalence resulted in theoretical numbers that were too low for using asymptotic chi-square validity test. Therefore, Fisher’s exact test (with Monte Carlo simulations) was used to test the independence between genotypes and indexing status for each virus species. The test was highly significant for BSOLV and BSGFV (Table 1). Examination of the contributions per cell showed that the response patterns of the genotypes were different depending on the virus species (Table 1). For BSOLV, the observed counts for positive indexations were significantly more numerous than expected for the Figue Pomme group, and less numerous for plantains. For BSGFV, the counts for positive indexations were significantly higher than expected for plantains, and lower for Figue Pomme and Cavendish (Table 1). Prevalence of BSOLV and BSGFV in the Cavendish cultivar was 0.5% and nil, respectively. The genome of this cultivar is devoid of infectious eBSVs therefore contaminations can occur solely through vector-borne transmission. Likewise, the prevalence of BSGFV in the Figue Pomme cultivar, whose genome is devoid of infectious eBSGFV allele GF7, was also nil. These results and the very low rate of infestation of sampled plants by mealybugs (7.1%; Supplementary Table 1) suggest that vector-borne transmission of BSVs by mealybugs occurs at very low rates in Guadeloupe. BSOLV and BSGFV infections were registered in varieties with AAB and ABB genomes harboring infectious eBSV alleles OL1 and GF7. Prevalence remained low, not exceeding 2.9%, and varied between cultivars (Table 1). In cultivar French Clair, prevalence of BSGFV (2.7%) was higher than that of BSOLV (0.2%), suggesting that infectious alleles GF7 and OL1 of this cultivar are differentially activated in the field. Likewise, in cultivar Figue Pomme, the prevalence of BSOLV (2.9%) was higher than that of BSGFV (0%), suggesting that the modified eBSOLV allele and allele GF7 of this cultivar are also differentially activated in the field. Infections were observed in samples of Poteau Géant, the only cooking banana cultivar with an ABB genotype that is cultivated in Guadeloupe. Overall, our results suggest that BSOLV and BSGFV infections monitored in Guadeloupe in triploid cultivars with an AAB genome, French Clair and Figue Pomme, and, to a lesser extent, in the triploid cultivar with an ABB genome Poteau Géant, resulted mainly from the activation of eBSOLV and eBSGFV infectious alleles under field conditions. A similar situation was reported previously in the Dominican Republic for triploid (AAB) hybrid cultivars Macho x Hembra and tetraploid (AAAB) hybrid FHIA 21, which also bear infectious alleles OL1 and GF7 (Martinez et al., 2020). Our results also suggest that the modified eBSOLV allele of cultivar Figue Pomme is infectious.

A comprehensive range of criteria was registered for each sample, including altitude, presence of BSV symptoms and presence of mealybugs. However, no statistically-supported correlation could be established between these criteria and infections by BSOLV or BSGFV. In particular, no correlation could be established between the presence of BSV symptoms such as leaf streaks, pseudostem splitting, cigar or petiole necrosis and BSV infections because none of the sampled plants displayed symptoms. Likewise, no mealybug was found on plants indexed positive at the time of sampling, although mealybugs were found on several non-infected plants (Supplementary Table 1). Similar observations about the scarcity or absence of symptoms in BSV-infected plants were made during previous surveys carried out in Australia (Daniells et al., 2001), Guadeloupe (Péréfarres et al., 2009), Cuba (Javer-Higginson et al., 2014) and the Dominican Republic (Martinez et al., 2020), suggesting that the impact of BSV infections is limited.

### Activation of Infectious eBSVs by Cell Culture

Cell culture is the most widely used method for multiplying vegetatively propagated crops such as banana. However, this technique has been shown to trigger the activation of eBSV infectious alleles in several interspecific banana hybrid varieties with AAB or AAAB genotypes created by breeding programs (Dallot et al., 2001; Lheureux et al., 2003; Côte et al., 2010) and in two plantains with an AAB genome, Kelong mekintu and Black Penkelon (Côte et al., 2010). We investigated the activation of eBSOLV, eBSGFV and eBSIMV infectious alleles by cell culture over 10 subculture cycles in another nine interspecific cultivars, which were selected because they cover a wide range of genotypes and eBSV allelic patterns (Supp.Table 2). This selection includes plantain French Clair (FRC), a natural interspecific hybrid of the French group with an AAB genotype which is widely grown in Guadeloupe for the local market (Lassoudière, 2010; Supplementary Table 2); Poteau Géant (PGE), Pelipita (PLP) and Burro Cemsa (BRC), three cooking type bananas with ABB genomes of which only the former is somehow cultivated in Guadeloupe; Flhorban 914, a dessert banana hybrid with an AAB genome originating from CIRAD’s breeding program and not cultivated in Guadeloupe; and three cultivars maintained in the BRC TP with AB (Kunnan diploid; KND), AABB (Kunnan tetraploid; KNT) and BB (Pisang Klutuk Wulung; PKW and Pisang batu; PBT) genotypes. Cultivars Flhorban 902 and IDN tetraploid (IDT), which have AAA and AAAA genomes, respectively, were used as negative controls since they are devoid of eBSVs (Duroy et al., 2016) whereas cultivar Kelong mekintu (KMT), a plantain with an AAB genotype, was included as a reference because it was assessed previously in a similar experiment (Côte et al., 2010).

**FIGURE 1:**
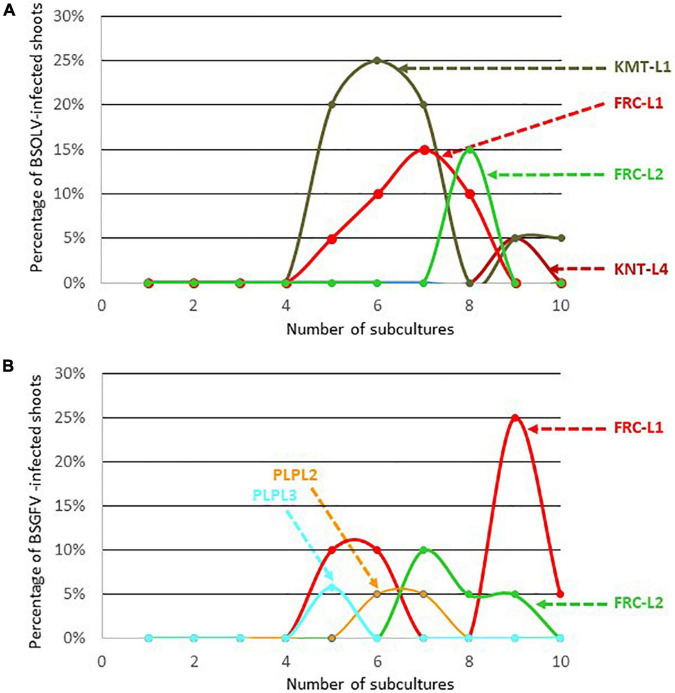
Kinetics of activation of eBSOLV **(A)** and eBSGFV **(B)** infectious alleles in cultivars Kelong mekintu (KMT), French Clair (FRC), Kunnan tetraploid (KNT) and Pelipita (PLP) during cell culture.

All the explants used as starting material for cell culture originated from plants that were negatively indexed for all the viruses known to infect bananas, including BSOLV, BSGFV, and BSIMV. They were virus-free at the beginning of the experiment, therefore infections by BSOLV, BSGFV, and BSIMV in plantlets originating from these explants could result solely from the activation of infectious eBSV alleles. The percentage of plantlets indexed positive for these three BSV species was considered the percentage of activation of the corresponding infectious allele, as in similar previous studies (Dallot et al., 2001; Côte et al., 2010). No plantlet was indexed positive for BSIMV throughout the experiment, especially for cultivars Flhorban 914, Poteau Géant, Burro Cemsa, Pélipita, Pisang Klutuk Wulung and Pisang batu, which harbor infectious allele IM (Supplementary Table 2). This result suggests that infectious allele IM in these cultivars is not activated by cell culture. On the contrary, activation of the infectious allele OL1 occurred in cultivars French Clair and Kelong mekintu (Figure 1A and Supplementary Table 3) whereas activation of infectious allele GF7 occurred only in cultivar French Clair (Figure 1B and Supplementary Table 3). Activation of eBSOLV and eBSGFV modified alleles was registered in cultivars Kunnan tetraploid and Pelipita, respectively, showing that these modified alleles are infectious (Figures 1A,B and Supplementary Table 3). Figures 1A,B show that activation of eBSOLV and eBSGFV infectious alleles occurred in these cultivars after a lag of several subcultures as similarly observed by Côte et al. (2010) for three plantain cultivars, including Kelong mekintu. Activation of infectious eBSOLV allele OL1 increased sharply after the first four subcultures for cultivar Kelong mekintu, as previously reported by Côte et al. (2010), and for line L1 of cultivar French Clair, reaching values of 25% at subculture #6 and 15% at subculture #7, respectively (Figure 1A). These values decreased sharply afterwards and zeroed after one to two additional subcultures. Residual activation was monitored for cultivar Kelong mekintu and plateaued at 5% at subcultures 9 and 10 (Figure 1). A similar pattern was observed for line 2 of cultivar French Clair (FRC-L2) and for line 4 of cultivar Kunnan tetraploid (KNT-L4), although in their case activation occurred later in the experiment (after subcultures 8 and 9, respectively), reached lower maximal values and did not rise again after zeroing. Activation of eBSGFV infectious alleles occurred in cultivars French Clair and Pelipita (Figure 1B and Supplementary Table 3). Here again, no activation was registered before subculture #4 and maximal activation rates differed substantially between cultivars (6% for Pelipita and 25% for French Clair). Maximal activation rates also differed between lines L1 (25%) and L2 (10%) of cultivar French Clair, respectively. Interestingly, Côte et al. (2010) reported that distinct lines of cultivar Kelong mekintu also reached different maximal activation rates at distinct subculture steps. On the contrary, very similar maximal activation rates of eBSGFV infectious alleles were registered for lines L2 and L3 of cultivar Pelipita (5 and 6%, respectively).

Modified eBSOLV alleles are present in the genome of cultivars Kunnan tetraploid, Kunnan diploid, Poteau Géant and Burro Cemsa whereas modified eBSGFV alleles are present in the genomes of cultivars Kelong mekintu and Pelipita (Supplementary Table 2). Activation of modified eBSOLV alleles occurred in Kunnan tetraploid but not in Kunnan diploid, Poteau Géant and Burro Cemsa (Figure 1A and Supplementary Table 3). Likewise, activation of modified eBSGFV alleles occurred in Pelipita but not in Kelong mekintu (Supplementary Table 3), confirming previous findings (Côte et al., 2010).

No activation of infectious alleles OL1, GF7, and IM was monitored in cultivars Pisang Klutuk Wulung and Pisang batu with diploid *M. balbisiana* genomes, supporting the hypothesis that the expression of infectious eBSVs is repressed in *M. balbisiana* diploids (Duroy et al., 2016).

### Activation of Infectious eBSVs During Field Culture

**FIGURE 2:**
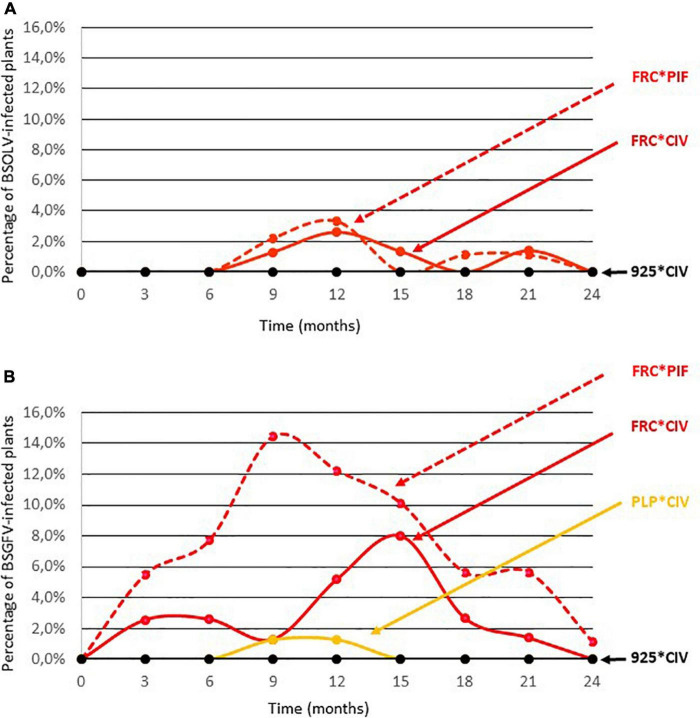
Kinetics of activation of eBSOLV **(A)** and eBSGFV **(B)** infectious alleles during the field trial in plants of cultivars French Clair (FRC) and Pelipita (PLP) originating from cell culture (*VP) or horticultural multiplication (*PIF). Cultivar Flhorban 925 was used as a negative control of activation.

The activation of eBSOLV, eBSGFV and eBSIMV infectious alleles under field conditions was monitored in cultivars French Clair and Pelipita. Cultivar French Clair was chosen because it is widely grown in Guadeloupe and because its infectious eBSOLV and eBSGFV alleles are activated by cell culture, raising concerns about the ability of this cultivar to promote BSV outbreaks under field cultivation conditions in Guadeloupe. Cultivar Pelipita was chosen for similar reasons, although it is not cultivated in Guadeloupe but was introduced from the Philippines into Latin America where it has become popular. Two types of planting materials were used for both cultivars to assess the impact of plant production mode on the activation of infectious eBSVs in the field: vitroplants produced by cell culture and plantlets produced by horticultural multiplication. An experimental plot was planned in 80 randomized complete blocks of five plants each (Supplementary Table 4 and Supplementary Figure 2). Due to shortage of planting material produced for some cultivar*mode of multiplication combinations, only 66 of the 80 blocks were complete and contained one of each type of planting material (French Clair vitroplants, FRC*VP; French Clair PIF, FRC*PIF; Pelipita vitroplants, PLP*VP; Pelipita PIF, PLP*PIF; Flhorban 925 vitroplants, 925*VP). In 11 blocks, the PLP*PIF combination had to be replaced by the FRC*PIF combination whereas in another 3 blocks, the PLP*VP and/or the FRC*PIF combinations had to be replaced by the 925*VP combination (Supplementary Figure 2 and Supplementary Table 4). The plot was surrounded by an external border of plants of the 925*VP combination. The field trial was carried out during two to three crop cycles, depending on varieties, spanning 24 months. Standard cultivation practices were implemented over the duration of the trial, including bunch harvest at the end of each production cycle, and desuckering, meaning that only one sucker was maintained to replace the mother plant after it was cut at the end of a production cycle while all other suckers were eliminated. All the plants produced for this experiment were indexed for BSOLV, BSGFV, and BSIMV at the end of the weaning stage. Results showed that activation of eBSOLV and eBSGFV infectious alleles occurred in one French Clair plant originating from cell culture and in four French Clair plants originating from horticultural multiplication, respectively, resulting in activation rates of 1.25 and 2.84%, respectively (Supplementary Table 6). No activation was recorded for the other types of planting material. Infected plants were discarded and only virus-free plants were used for planting. Figure 2 provides a graphical representation of the activation dynamics of eBSOLV and eBSGFV infectious alleles during the experiment. None of the Flhorban 925 plants tested positive throughout the experiment, showing that vector-borne transmission did not occur and that registered infections resulted solely from the activation of infectious alleles. No infection by BSIMV occurred in cultivar Pelipita which harbors the IM infectious allele, showing that activation of this infectious allele was not triggered by the cultivation conditions of the field trial. On the contrary, activation of infectious eBSOLV and eBSGFV alleles was registered. In cultivar French Clair, maximal activation rates reached 3.3% for infectious allele OL1 (Figure 2A and Supplementary Table 7B) and 14.4% for infectious allele GF7 (Figure 2B and Supplementary Table 7D), respectively, and were registered in plants originating from horticultural multiplication. Maximum activation rates of infectious alleles OL1 and GF7 in French Clair plants originating from cell culture were 2.6% and 8%, respectively (Figures 2A,B and Supplementary Tables 7B,D). Activation of OL1 started at the beginning of the experiment whereas that of GF7 started 6 months later. These results suggest that the infectious alleles OL1 and GF7 of cultivar French Clair are differentially triggered by abiotic stresses occurring under field cultivation conditions and that activation is stronger in plants originating from horticultural multiplication than in plants originating from cell culture, especially for infectious allele GF7. The kinetics of activation of OL1 and GF7 in French Clair plants originating from cell culture was similar to that observed for this cultivar during cell culture, with a first increase followed by a decrease and a rebound in activation rates (Figures 1B,C). Maximal activation rate in French Clair was higher for infectious allele GF7 (14.4%) than for infectious allele OL1 (3.3%; Figure 2 and Supplementary Tables 7B,D). Activation of modified eBSGFV allele occurred in plants of cultivar Pelipita originating from cell culture, at a low maximal rate (1.3%; Figure 2B and Supplementary Table 7D). Here again, the kinetics of activation of modified eBSGFV allele in Pelipita during the field trial was similar to that observed for this cultivar during cell culture (Figure 1C). No activation of eBSGFV modified allele was registered in Pelipita plants originating from horticultural multiplication (Figure 2B and Supplementary Table 7D). Likewise, no activation of infectious allele OL1 was registered in Pelipita plants originating from either cell culture or horticultural multiplication, as also observed during cell culture (Figure 1A and Supplementary Table 3B). Activation of eBSOLV and eBSGFV infectious alleles decreased over time and zeroed by the end of the experiment, except in one French Clair plant originating from horticultural multiplication which remained infected by BSGFV at this stage. Some plants were repeatedly indexed positive over several consecutive sampling timepoints whereas others were indexed positive at non-consecutive timepoints or only once (Supplementary Figure 3). Over the duration of the experiment, only one BSOLV-infected plant (FRC*VP plant N°12; Supplementary Table 4) briefly displayed mild leaf streak symptoms at month 21. All the other plants of the field trial remained symptomless throughout the experiment and no impact of BSOLV and BSGFV infections on plant growth, fruit yield and fruit quality were noticed. For statistical analyses, data were first filtered by excluding the 14 plants that died during the experiment and five blocks, either because these blocks had less than three treatments (blocks 78, 79, and 80), or because two plants in the same block died (blocks 49 and 55; Supplementary Table 4). Treatment T5, which was included in the design to control the absence of external viral contamination, and whose mean was zero, was not considered in the analyses. Since many blocks had zero mean and variance, the block factor was treated as a random factor. Two generalized mixed models were tested: a 2 × 2 factorial model with interaction, and a one-way model with 4 treatments, for BSOLV and BSGFV, at 9 and 12 months (SAS 9.3 software, proc GLIMMIX). None of the models succeeded in estimating the effects of factors on activation rates because at least one of the treatments had a zero mean, and the algorithm did not converge.

The activation of infectious alleles OL1 and GF7 in cultivar French Clair by field culture was further assessed at a larger scale in nine plots totaling 3,123 plants originating from vitroplants (Supplementary Figure 4 and Supplementary Table 5). Since the genome of cultivar French Clair does not harbor infectious allele IM, only activation of infectious alleles OL1 and GF7 were monitored. Viral indexings were performed at the end of the weaning stage on 10% of the French Clair vitroplants produced for planting: 14.3% of the plants were found infected by BSOLV and 1.3% by BSGFV. Infected plants were discarded; however, it is likely that similar proportions of infected plants were present in the planting material used in the plots, reflecting the situation that farmers would encounter when using French Clair plants originating from vitroplants. Plant mortality occurred in all the plots between planting and the first sampling, resulting in an overall decrease in the number of plants of the experiment. On the contrary, the number of plants of plots 2, 3, 5, 7, and 9 increased between sampling 1 at month 12 and sampling 2 at month 24, because some farmers attempted to restore plant numbers by purposely not performing a comprehensive desuckering after bunch harvest, as recommended by standard practices (Lassoudière, 2010), resulting in more than one sucker being retained for some plants in these plots. As a consequence, the overall number of plants of the experiment increased from 2,621 to 2,699 between sampling 1 and sampling 2 and plant densities were more variable between plots than initially planned, ranging between 1,107 and 1,822 plants per hectare at month 12 and between 1,006 and 1,795 plants per hectare at month 24 whereas they ranged between 1767 and 1,847 plants per hectare at planting time (Supplementary Table 5).

**FIGURE 3:**
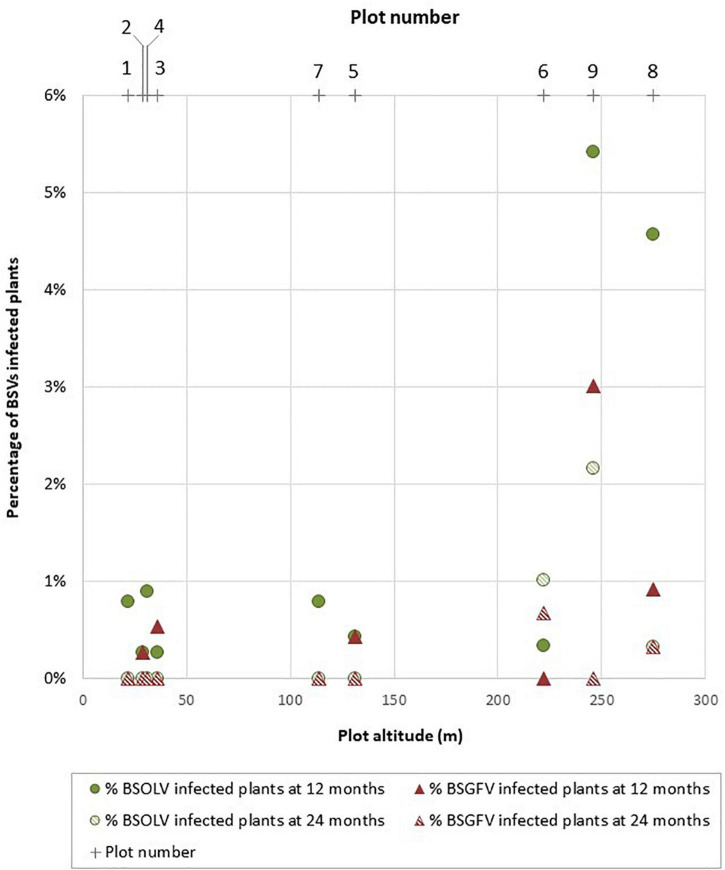
Activation patterns of eBSOLV and eBSGFV infectious alleles in cultivar French Clair during a multi-site trial. Plot numbers and plot altitudes are shown.

Activation rates varied between plots for both OL1 and GF7 infectious alleles, suggesting that criteria that vary between these plots, such as farming practices or altitude, may influence activation (Figure 3). The relationship between the rate of infected plants as a function of altitude is not linear and therefore does not allow a reasonable linear correlation to be calculated (Figure 3). However, the shape of the relationship is structured. In low and medium altitude plots (<150 m), infection rates remained below 1% throughout the duration of the experiment for both BSOLV and BSGFV whereas the highest rates of infected plants (>1%) were only observed in high altitude plots (≥ 250 m; plots 8 and 9; Supplementary Table 5 and Figure 3). Beyond an interpretation of these results as a simple “plot effect”, the shape of the scatterplot suggests a threshold effect for climatic stresses, such as cumulative rainfall or day/night temperature differences, which altitude only roughly reflects. Activation rates at 12 months were less than 1% (Supplementary Table 5) except in plots 8 (4.6% for OL1) and 9 (6% for OL1 and 3% for GF7). Activation rates were even lower at month 24, ranging between 0 and 0.4%, except for plot 6 where OL1 and GF7 activation rates increased from 0.3 to 1% and from 0 to 0.7%, respectively, between month 12 and month 24 (Supplementary Table 5). Overall, these results are consistent with the trend observed in the smaller-scaled field trial described above (Figure 2) and confirmed that activation of eBSVs infectious alleles decreases over time. Here again, only one plant showed mild leaf symptoms at 12 months and no impact of BSOLV and BSGFV infections on plant growth, fruit yield and production could be observed throughout the experiment.

## Discussion

*Caulimoviridae* endogenous viral elements (EVEs) are widespread in plant genomes and most of them are non-infectious (Teycheney and Geering, 2011; Geering et al., 2014; Diop et al., 2018). However, infectious alleles of endogenous banana streak viruses (eBSVs) are present in the genome of banana interspecific hybrid cultivars combining *M. acuminata* (A) and *M. balbisiana* (B) genomes and hybridization is known to activate the expression of EVEs in plants (Lopez-Gomollon et al., 2022). Indeed, the expression of infectious eBSV alleles occurs in banana interspecific hybrid cultivars. Activation is triggered by biotic and abiotic stresses and leads to spontaneous infections by cognate viruses, posing a potential risk of large-scale dissemination of BSVs if activation is coupled with vector-borne transmission of the viral particles (Meyer et al., 2008). Activation during vegetative propagation by the cell culture techniques used to mass-produce vitroplants has been reported previously and quantified in four interspecific cultivars (Dallot et al., 2001; Côte et al., 2010) whereas activation during culture in the field has been regularly observed or suspected but never assessed or quantified. In this work, we implemented a comprehensive approach to BSV risk assessment in several interspecific cultivars with various genotypes at the scale of a tropical island territory, Guadeloupe.

A prevalence study of BSOLV, BSGFV, and BSIMV species, for which infectious eBSVs are widespread in *M. balbisiana* genomes, was carried out in Guadeloupe, using a sampling representative of the main banana cultivation areas and cultivars that have been grown for decades. The results of this large-scale survey confirmed the absence of BSIMV from Guadeloupe that was reported previously from a smaller sampling (Péréfarres et al., 2009). They also suggest that transmission of BSOLV and BSGFV species by mealybugs is marginal in Guadeloupe. This situation might result from BSVs’ main mealybug vector species (*Planococcus citri*, *P. minor*, *Dysmycoccus brevipes*, *Saccharicoccus sacchari*) being scarce on banana in Guadeloupe, although they are widespread on various other crops (Matile-Ferrero and Etienne, 2006; Supplementary Table 1) and from low transmission rates of badnaviruses by mealybugs (Kirkpatrick, 1950; Hull, 2002). A similar situation was reported previously in Cuba and the Dominican Republic (Javer-Higginson et al., 2014; Martinez et al., 2020), suggesting that low rates of mealybug transmission of BSVs may be a general trend in Caribbean island biotopes. BSOLV and BSGFV infection rates in samples originating from plants with AAB genotypes (French Clair and Figue Pomme) increased with altitude of the sampling sites (Supplementary Figure 5), suggesting that abiotic factors changing with altitude, such as rainfalls and/or day/night temperature differences, are associated with variations in prevalence.

We monitored the activation of infectious eBSOLV and eBSGFV alleles by cell culture in cultivars of various genotypes. The multiplication process started from a single explant. Therefore, only low numbers of shoots were produced for all lines of all cultivars during the first four subcultures, as expected. Moreover, cultivars and even suckers of a given cultivar displayed different proliferation rates, resulting in differences in the numbers of plantlets produced after a given number of subcultures. All these factors limited the number of samples available for viral indexings (Supplementary Table 3) leading to low accuracy of the percentages of infected plants during the early stages of the experiment. From the fifth subculture onwards, the numbers of produced shoots available for indexing increased, resulting in a better accuracy. Activation occurred during cell culture for infectious allele OL1 in cultivars French Clair and Kelong mekintu and for infectious allele GF7 in cultivar French Clair (Supplementary Table 3 and Figure 1). Activation rates differed by up to a factor 5 for OL1 and up to a factor 4 for GF7 between cultivars and even between lines of a given cultivar, suggesting that complex interactions between genetic and environment factors are involved in activation and modulate it. Activation of modified eBSOLV allele occurred during cell culture in cultivar Kunnan tetraploid (KNT-L4) whereas that of modified eBSGFV allele occurred in Pelipita (PLP-L2 and PLP-L3), providing evidence that these modified alleles are infectious and cause infections upon activation, and that modified eBSOLV and eBSGFV alleles should not be overlooked when analyzing eBSV molecular patterns. A similar conclusion can be drawn from the BSOLV infections detected in the samples of cultivar Figue pomme collected during the prevalence survey (Table 1), since this cultivar also hosts a modified eBSOLV allele. On the contrary, no activation of modified eBSOLV allele was registered in cultivar Poteau Géant during cell culture although BSOLV-infected samples of this cultivar were identified during the prevalence survey, suggesting that Poteau Géant’s modified eBSOLV allele is infectious and that it is activated by abiotic stresses resulting from field culture but not by abiotic stresses resulting from cell culture. It has been hypothesized that the expression of infectious eBSV alleles is repressed in *M. balbisiana* diploids (Duroy et al., 2016). However, BSOLV and BSGFV infections monitored during cell culture in cultivars Kunnan T (AABB) and Pelipita (ABB), respectively, show that such a repression does not occur in these interspecific cultivars whose genome carry two copies of the B genome. This situation could result from genetic factors brought by the *M. acuminata* genome lifting mechanisms repressing the expression of infectious eBSVs, or from these AABB and ABB genomes not being true *M. balbisiana* diploids at the insertion loci of infectious eBSVs, following interspecific recombination between A and B chromosomes during the hybridization process (Baurens et al., 2019). We observed a decrease in activation levels over time during cell culture (Figure 1) that is similar to that observed by Côte et al. (2010) in a more limited range of interspecific hybrid cultivars. Our findings support the hypothesis proposed by these authors that cell multiplication in neoformed plantlets outcompetes virus replication resulting from the activation of infectious eBSV alleles, resulting in the observed decrease in activation levels over time. They also provide evidence that this phenomenon is likely to occur in all interspecific cultivars with AAB and AAAB genotypes and that extending the number of subcultures should be recommended to minimize the risk of producing BSOLV- or BSGFV-infected vitroplants when multiplying such genotypes.

Cultivar French Clair is grown over tens of hectares throughout Guadeloupe where there is an increasing demand for supplying growers with vitroplants in order to limit the spread of nematodes, weevils, bacterial and viral diseases associated with the use of non-certified planting material such as suckers. This situation prompted a large-scale study of the activation of infectious alleles OL1 and GF7 in cultivar French Clair under field culture conditions in Guadeloupe. A field trial showed that infectious alleles OL1 and GF7 are expressed in cultivar French Clair under field conditions, leading to asymptomatic infections, and that activation decreases over time. We found that infectious allele GF7 of cultivar French Clair was more prone to activation in plants originating from horticultural multiplication than in plants originating from vitroplants, whereas activation levels were similar for infectious allele OL1 in both types of plants. A larger scale multi-site study confirmed that activation of OL1 and GF7 occurs under field conditions in French Clair plants originating from vitroplants and causes symptomless infections with no visible impact on plant growth, fruit yield and fruit quality in Guadeloupe. Overall, our results show that the use of French Clair vitroplants as planting material is unlikely to cause BSV outbreaks in Guadeloupe and should be preferred to that of plantlets produced by horticultural multiplication in order to lower the risk of activation of eBSV infectious alleles during field culture.

## Data Availability Statement

The original contributions presented in the study are included in the article/Supplementary Material, further inquiries can be directed to the corresponding author/s.

## Author Contributions

MU, CD, and P-YT conceived the study. MU, GP, GF, KPP, CG, FL, J-PP, BJ, J-MD, and FS performed the experiments. MU, CD, and P-YT analyzed the data. P-YT drafted the manuscript. All authors contributed to the article and approved the submitted version.

## Conflict of Interest

The authors declare that the research was conducted in the absence of any commercial or financial relationships that could be construed as a potential conflict of interest.

## Publisher’s Note

All claims expressed in this article are solely those of the authors and do not necessarily represent those of their affiliated organizations, or those of the publisher, the editors and the reviewers. Any product that may be evaluated in this article, or claim that may be made by its manufacturer, is not guaranteed or endorsed by the publisher.

## Footnotes

1
http://collections.antilles.inra.fr/

## Supplementary Material

Supplementary Figure 1 Location of the collection sites used for the prevalence survey. Blue, red and gray triangles represent locations where BSGFV-, BSOLV- and non-infected samples were collected, respectively.

Supplementary Figure 2 Map of the experimental plot used to study the activation of eBSOLV and eBSGFV infectious alleles under field conditions in cultivars French Clair and Pelipita. Random blocks are delimited by thick lanes. Cultivar*multiplication mode combinations that were used to replace missing combinations are shown in red. Color codes used for each combination are the same as those used in Supplementary Table 4.

Supplementary Figure 3 Kinetics of activation of eBSOLV and eBSGFV infectious alleles in cultivars French Clair and Pelipita measured over a 24 months period in the experimental plot. Color codes used for each combination are the same as those used in Supplementary Table 4. BSV species detected in plants are specified: OL (BSOLV) and GF (BSGFV). **(A)** 3 months after planting; **(B)** 6 months after planting; **(C)** 9 months after planting; **(D)** 12 months after planting; **(E)** 15 months after planting; **(F)** 18 months after planting; **(G)** 21 months after planting; (H) 24 months after planting.

Supplementary Figure 4 Location of the experimental plots used for the multi-site assessment of the activation of infectious alleles OL1 and GF7 in cultivar French Clair.

Supplementary Figure 5 Percentage of BSOLV- and BSGFV-infected samples collected during the prevalence survey from plants with an AAB genotype (French Clair, Figue pomme). Percentages of infected samples were distributed according to the altitude of the sampling sites.
