# Supplementary figures and images for: Risk Assessment of Infectious Endogenous Banana Streak Viruses in Guadeloupe
Source: Front Plant Sci. 2022 Jul 11;13:951285. doi: 10.3389/fpls.2022.951285 (PMC9310019; doi:10.3389/fpls.2022.951285)

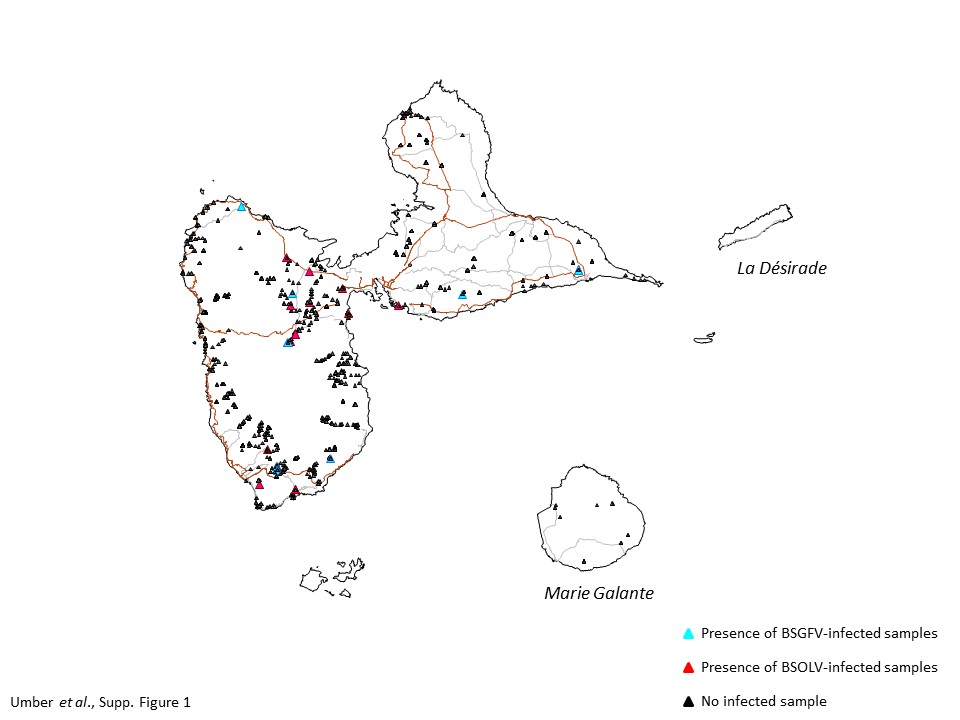

Supplement: Supplementary Figure 1 — Location of the collection sites used for the prevalence survey. Blue, red and gray triangles represent locations where BSGFV-, BSOLV- and non-infected samples were collected, respectively. [file Image_1.jpg]

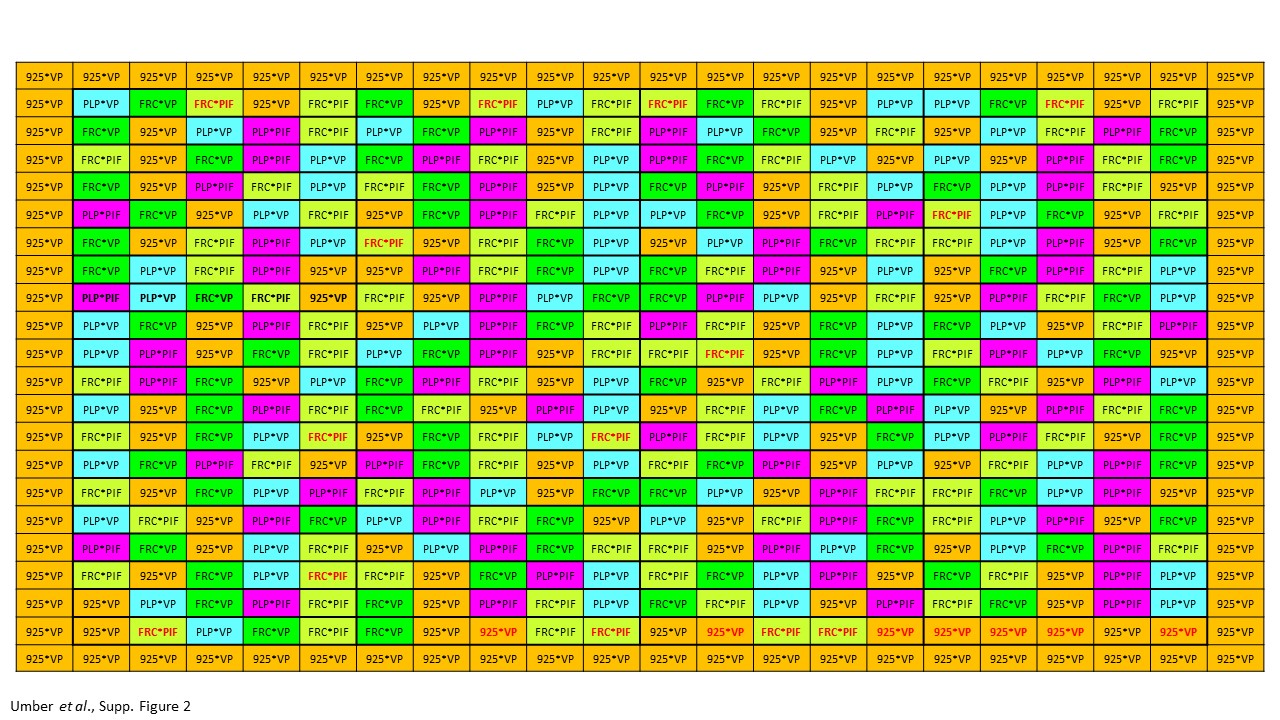

Supplement: Supplementary Figure 2 — Map of the experimental plot used to study the activation of eBSOLV and eBSGFV infectious alleles under field conditions in cultivars French Clair and Pelipita. Random blocks are delimited by thick lanes. Cultivar*multiplication mode combinations that were used to replace missing combinations are shown in red. Color codes used for each combination are the same as those used in Supplementary Table 4. [file Image_2.jpg]

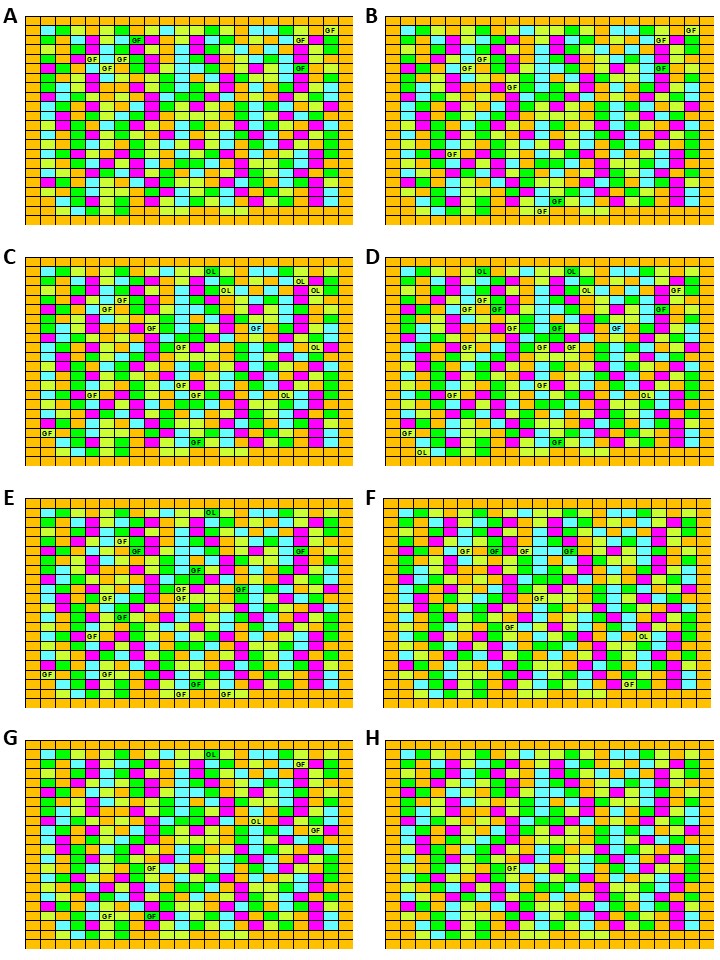

Supplement: Supplementary Figure 3 — Kinetics of activation of eBSOLV and eBSGFV infectious alleles in cultivars French Clair and Pelipita measured over a 24 months period in the experimental plot. Color codes used for each combination are the same as those used in Supplementary Table 4. BSV species detected in plants are specified: OL (BSOLV) and GF (BSGFV). (A) 3 months after planting; (B) 6 months after planting; (C) 9 months after planting; (D) 12 months after planting; (E) 15 months after planting; (F) 18 months after planting; (G) 21 months after planting; (H) 24 months after planting. [file Image_3.jpg]

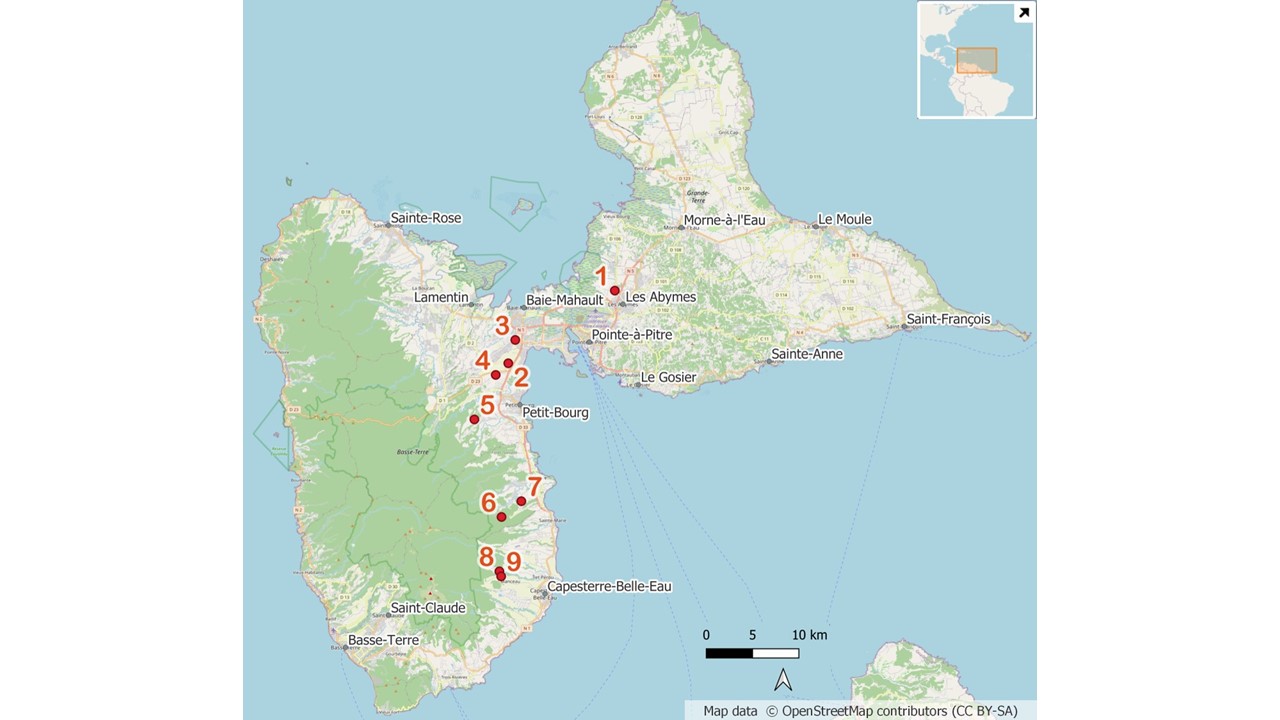

Supplement: Supplementary Figure 4 — Location of the experimental plots used for the multi-site assessment of the activation of infectious alleles OL1 and GF7 in cultivar French Clair. [file Image_4.jpg]

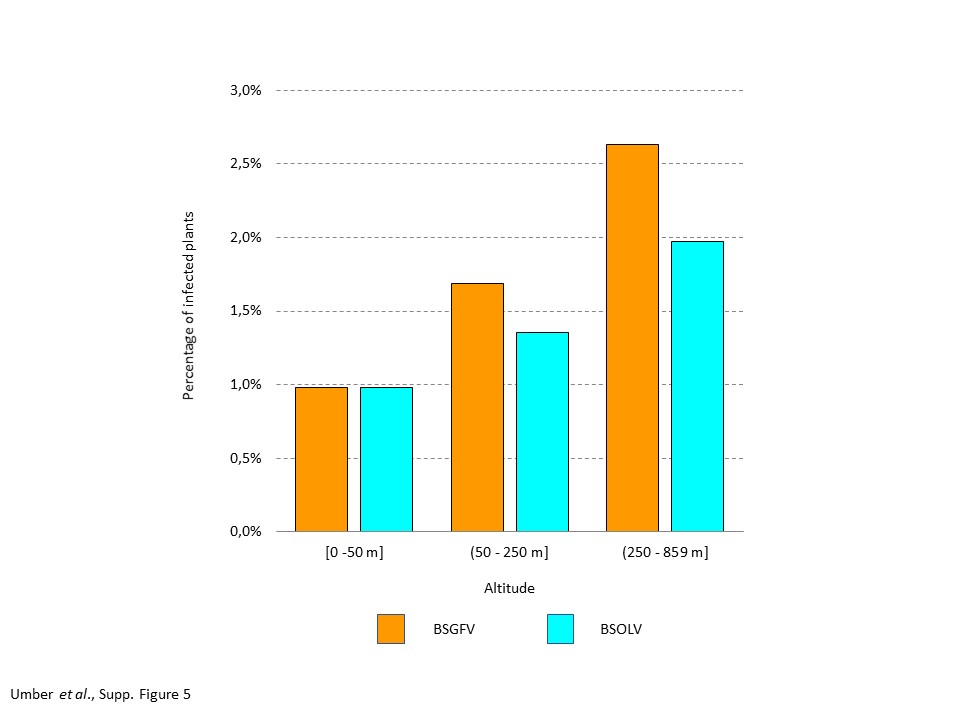

Supplement: Supplementary Figure 5 — Percentage of BSOLV- and BSGFV-infected samples collected during the prevalence survey from plants with an AAB genotype (French Clair, Figue pomme). Percentages of infected samples were distributed according to the altitude of the sampling sites. [file Image_5.jpg]
